# The Role of Curcumin in Prostate Cancer Cells and Derived Spheroids

**DOI:** 10.3390/cancers14143348

**Published:** 2022-07-09

**Authors:** Mariarosaria Boccellino, Pasqualina Ambrosio, Andrea Ballini, Danila De Vito, Salvatore Scacco, Stefania Cantore, Antonia Feola, Marzia Di Donato, Lucio Quagliuolo, Antonella Sciarra, Giovanni Galasso, Felice Crocetto, Ciro Imbimbo, Silvia Boffo, Erika Di Zazzo, Marina Di Domenico

**Affiliations:** 1 Department of Precision Medicine, University of Campania “Luigi Vanvitelli”, 80138 Naples, Italy; mariarosaria.boccellino@unicampania.it (M.B.); pasqualina.ambrosio@unicampania.it (P.A.); tonia.feola@gmail.com (A.F.); marzia.didonato@unicampania.it (M.D.D.); lucio.quagliuolo@unicampania.it (L.Q.); giovanni.galasso@unicampania.it (G.G.); erika.dizazzo@unimol.it (E.D.Z.); marina.didomenico@unicampania.it (M.D.D.); 2 Department of Basic Medical Sciences, Neurosciences and Sense Organs, University of Bari “Aldo Moro”, 70124 Bari, Italy; danila.devito@uniba.it (D.D.V.); salvatore.scacco@uniba.it (S.S.); 3 Independent Researcher, 70129 Bari, Italy; 4 Department of Biology, University of Naples “Federico II”, 80126 Naples, Italy; antonella.sciarra@unicampania.it; 5 Department of Neuroscience, Reproductive Sciences and Dentistry, School of Medicine, University of Naples “Federico II”, 80131 Naples, Italy; felice.crocetto@gmail.com (F.C.); ciro.imbimbo@unina.it (C.I.); 6 Department of Biology, College of Science and Technology, Temple University, Philadelphia, PA 19122-6078, USA; silvia.boffo@temple.edu

**Keywords:** prostate cancer, metastasis, curcumin, chemotherapeutic agents, 3D cell culture, spheroids

## Abstract

**Simple Summary:**

In recent years, considerable efforts have been made to discover new compounds useful for prostate cancer (PC) therapy and related clinical management. Promising advances in this field were reached with nutraceutical compounds. Curcumin represents an attractive therapeutic agent for mCRPC patients. Curcumin treatment displayed a dose-dependent reduction of DU145 and PC-3 PC cell viability similar to chemotherapeutic agents (paclitaxel, cisplatin, and docetaxel). Furthermore, by exploring the EGFR-mediated signaling, we observed that the treatment with both chemotherapeutic agents and curcumin reduced EGFR expression levels and ERK activation. Finally, we demonstrated that curcumin reduces the size of DU145 and PC-3 3D cell spheroids and has the potential to induce apoptosis.

**Abstract:**

A major challenge in the clinical management of prostate cancer (PC) is to inhibit tumor growth and prevent metastatic spreading. In recent years, considerable efforts have been made to discover new compounds useful for PC therapy, and promising advances in this field were reached. Drugs currently used in PC therapy frequently induce resistance and PC progresses toward metastatic castration-resistant forms (mCRPC), making it virtually incurable. Curcumin, a commercially available nutritional supplement, represents an attractive therapeutic agent for mCRPC patients. In the present study, we compared the effects of chemotherapeutic drugs such as docetaxel, paclitaxel, and cisplatin, to curcumin, on two PC cell lines displaying a different metastatic potential: DU145 (moderate metastatic potential) and PC-3 (high metastatic potential). Our results revealed a dose-dependent reduction of DU145 and PC-3 cell viability upon treatment with curcumin similar to chemotherapeutic agents (paclitaxel, cisplatin, and docetaxel). Furthermore, we explored the EGFR-mediated signaling effects on ERK activation in DU145 and PC-3 cells. Our results showed that DU145 and PC-3 cells overexpress EGFR, and the treatment with chemotherapeutic agents or curcumin reduced EGFR expression levels and ERK activation. Finally, chemotherapeutic agents and curcumin reduced the size of DU145 and PC-3 spheroids and have the potential to induce apoptosis and also in Matrigel. In conclusion, despite different studies being carried out to identify the potential synergistic curcumin combinations with chemopreventive/therapeutic efficacy for inhibiting PC growth, the results show the ability of curcumin used alone, or in combinatorial approaches, to impair the size and the viability of PC-derived spheroids.

## 1. Introduction

Prostate cancer (PC) represents the most commonly diagnosed tumor in men and the second leading cause of male cancer-related deaths in Western society [1]. The early-stage treatments include radical prostatectomy, radiotherapy, brachytherapy, cryotherapy, and androgen deprivation therapy (ADT) [1,2,3,4,5,6,7,8,9,10]. At an early stage, PC is an androgen-dependent disease, and ADT represents the major therapeutic option. However, ADT frequently fails, and PC progresses to an androgen-independent state, also known as castration-resistant PC (CRPC), accomplished by a poor prognosis and high metastatic potential [11,12,13,14]. Currently, research is focused on the study of the molecular mechanisms underlying resistance to hormone-targeted therapies [15,16,17,18,19,20,21,22,23,24]. Metastatic spreading of CRPC (mCRPC) still represents the major cause of PC-related death and compounds, such as taxanes, poly (adenosine diphosphate-ribose) polymerase (PARP) inhibitors, and programmed cell death 1 (PD-1) inhibitors, have entered clinical trials [25]. In mCRPC, chemotherapy is used as a palliative to alleviate the symptoms and ensure a better quality of patient life. Cisplatin activates several signal transduction pathways, including ERK, p53, p73 and MAPK [26,27], which culminates in apoptosis activation. Although cisplatin represents one of the most widely used chemotherapeutics, it is associated with significant dose-limiting toxicities, including nephrotoxicity and neurotoxicity [28,29]. Consequently, it is necessary the development of more effective and safe therapeutic strategies [30]. Paclitaxel and docetaxel are taxoids used in different cancer types [31]. In particular, paclitaxel impairs VEGF-mediated angiogenesis, and [32] docetaxel represents the first chemotherapeutic agent able to prolong survival in mCRPC. However, patients with mCRPC often experience drug resistance, which limits its use [33,34,35,36,37,38,39,40,41,42,43,44]. Although, even if several therapeutic approaches for mCRPC treatment are available, the disease often remains incurable. Lifestyle plays a pivotal role in cancer: both a balanced diet and exercise might reduce the PC risk [45,46,47,48,49]. Nutraceuticals could reduce cancer incidence as suggested by epidemiologic and animal model studies. Curcumin (diferuloylmethane), a yellow pigment in the Indian spice turmeric (*Curcuma longa*), is endowed with antioxidant, anti-inflammatory, anticancer, antiviral, antibacterial and anti-diabetic properties. Curcumin was proposed for PC therapy [50,51,52,53,54]. The generation of in vitro 3D miniaturized models in the extracellular matrix (ECM) represents a significant approach that allows for more efficient test drugs in which diffusion could be inhibited by the ECM in vivo [55,56]. The 3D multicellular organoids established in vitro, mimicking the corresponding in vivo organ, can be used to recapitulate organ behavior in the tissue culture dish. This near-physiological 3D model promotes an accurate study of a range of physiological processes in vivo, such as tissue renewal and responses to damage and drugs.

**Figure 1:**
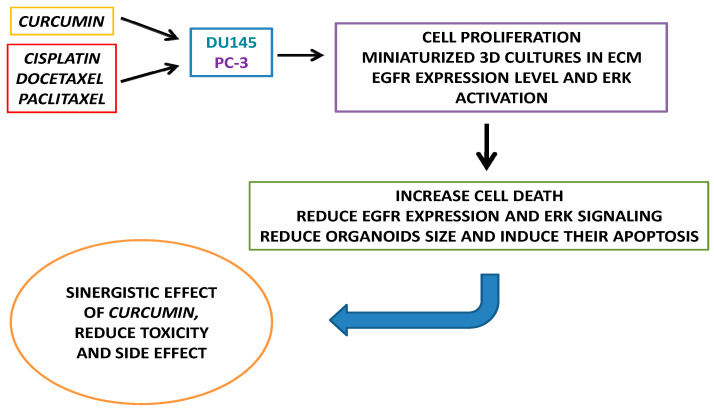
Schematic representation of the study workflow.

The aim of the present study was to evaluate the effects of curcumin alone or in combination with cisplatin (CDDP), paclitaxel (PTX), and docetaxel (DOC), on cell viability of two different metastatic PC cell lines and the growth and survival of PC-derived spheroids (Figure 1).

## 2. Materials and Methods

### 2.1. Chemicals and Reagents

Chemotherapeutic agents, such as cisplatin (Accord Healthcare, Milan, Italy), paclitaxel (Accord Healthcare, Milan, Italy), and docetaxel (Hospira, Naples, Italy), were dissolved in 0.9% NaCl and added to the culture medium at the concentrations indicated in Table 1. Curcumin from *Curcuma longa* (≥95% purity), was purchased from Sigma-Aldrich (St. Louis, MO, USA). A 10 mM stock solution was prepared in dimethylsulfoxide (DMSO, MP Biomedicals, Santa Ana, CA, USA) and stored at +4 °C.

### 2.2. Cell Cultures

Human DU145 and PC-3 PC cells were provided from American Type Culture Collection (Rockville, MD, USA). Cells were maintained at 37 °C in a humidified 5% carbon dioxide (CO_2_) atmosphere. DU145 cells were cultured in DMEM containing 10% fetal bovine serum (FBS), penicillin (100 U/mL), streptomycin (100 U/mL) and glutamine (2 mM).

PC-3 cells were cultured in phenol-red Roswell Park Memorial Institute (RPMI)-1640/F12, containing 10% FBS, penicillin (100 U/mL), streptomycin (100 U/mL) and glutamine (2 mM). Media and supplements were provided by Gibco (Thermofisher, Waltham, MA, USA). The cell lines were routinely monitored for Mycoplasma contamination. In our experiments, we used DU145 and PC-3 PC cells in passages 22 and 25, respectively.

### 2.3. Cell Survival Analysis

Growth inhibition was assessed by cell counting, and using the 3-(4,5-dimethylthiazol-2-yl)-2,5-biphenyltetrazolium bromide (MTT) (Sigma-Aldrich, Co., St. Louis, MO, USA) assays, as previously described [57,58]. Twenty-four hours (h) before stimulation, 3 × 10^3^ DU145 and PC-3 cells were plated in 96-well plates. Cells were treated for 48 and 72 h with eight different concentrations of cisplatin, paclitaxel, and docetaxel, and for 24, 48 and 72 h with six different concentrations of curcumin ranging between the values indicated in Table 1. IC50 values on DU145 and PC-3 cells were measured at 48 and 72 h, with Sigma Plot, as reported in Table 2.

### 2.4. Western Blotting Analysis

Cell lysates were prepared by treating cells with ice-cold lysis buffer (20 mM Tris pH 8.0, 1% NP40, 10% glycerol, 137 mM NaCl, 10 mM EDTA pH 8.0, and Roche Applied Science (Roche Applied Science, Monza, Italy) protease inhibitor cocktail “Complete”) for 20 min, followed by high-speed centrifugation at 4 °C for 15 min [59,60]. Proteins (50 µg) were separated on 12% SDS-PAGE gels and then transferred to polyvinylidene difluoride membrane.

The blots were blocked with 5% non-fat dry milk in 20 mM Tris/HCl, pH 7.5, 500 mM NaCl plus 0.1% Tween 20 (TBS-T). The membranes were subsequently incubated in agitation at 4 °C overnight in 1% BSA-TBS-T buffer containing the specific antibody against: p-ERK (Santa Cruz Biotechnology, Inc., Dallas, TX, USA), Erk 1/2 (cell Signaling cat n°9102 1:1000 3%BSA), EGFR (Santacruz sc-03 1:500 3%Milk O.N.) and tubulin (Santacruz sc-5286 1:1000 3%milk O.N.).

After washing four times with TBS-T, the blots were incubated for 1 h at room temperature (RT) with the second antibody conjugated to peroxidase, washed four times with TBS-T, developed with ECL detection reagents (Amersham, Little Chalfort, Buckinghamshire, UK) for 1 min and exposed to X-Omat film (Eastman Kodak Co., Rochester, NY, USA). The whole western blot figures can be found in the supplementary figures.

### 2.5. Miniaturized 3D Cultures in ECM

DU145 and PC-3 cells (4 × 104), were mixed with 250 µL of growth factor reduced basement membrane matrix Corning™ Matrigel™ (Fisher Scientific Italia, Rodano, Italy), for each well, and embedding method was used for establishing organoids in 24-well plate [55]. Organoid plating medium was made using phenol-red DMEM/F12 medium, containing 5% FBS, penicillin (100 U/mL), streptomycin (100 U/mL), 1× glutamax, 10 mM Hepes, B27 (50× stock solution), 1 M nicotinamide, 500 mM N-acetylcysteine and 10 µM Y-27632 (Millipore, Burlington, MA, USA). After 3 days, the organoid-plating medium was replaced with a similar medium without N-acetylcysteine and Y-27632. On the 4th day, organoids were untreated or treated with the indicated drugs and the medium was changed every 2 days. Different fields were analyzed and the relative organoid size was calculated and expressed as a fold increase over the organoid area, calculated after 4 days as previously reported [56].

### 2.6. Immunofluorescence (IF) Analysis of Organoids

Detection of apoptosis was performed using the in situ cell death detection kit, Fluorescein (Roche Applied Science, Monza, Italy). Organoids were fixed with 4% Paraformaldehyde for 1 h at RT, permeabilized with 0.2% Triton X-100 for 45 min at RT, and incubated overnight at 37 °C with the pre-mixed solution (enzyme solution-label solution) following manufacturer’s instructions. Different fields were analyzed and phase-contrast and immunofluorescence (IF) microscopy images, were acquired using DMIRB Leica (Leica Microsystems S.r.l., Varese, Italy) microscope equipped with C-Plan 40× or HCX PL Fluotar 63× objectives (Leica), a DFC 450C camera (Leica Microsystems S.r.l., Varese, Italy) and the Application Suite Software (Leica Microsystems S.r.l., Varese, Italy).

Representative images of three independent experiments are presented. Detection of proliferating cells was performed using the rabbit monoclonal antibody Ki-67 (DB35; Cell Signaling, Danvers, MA, USA). Organoids were washed with PBS-glycine, fixed with 4% Paraformaldehyde for 20 min at room temperature (RT), permeabilized with IF solution (0.2% Triton X-100, 0.1% BSA, 0.05% Tween 20 in PBS for 45 min, and blocked in IF blocking solution (10% fetal bovin serum in IF buffer). Organoids were stained with Ki-67 primary antibody dissolved 1:70 in IF buffer (for 5 h at 37 °C in a humid chamber).

After extensive washings with PBS-glycine, organoids were incubated for 1 h with diluted (1:200 in IF buffer) Texas red-conjugated affinity pure anti-rabbit IgG (Jackson ImmunoResearch Inc., West Baltimore Pike, West Grove, PA, USA) at 37 °C in a humid chamber. Different fields were analyzed and Phase-contrast and IF microscopy images were acquired using DMIRB Leica (Leica Microsystems S.r.l., Varese, Italy) microscope equipped with C-Plan 40× or HCX PL Fluotar 63× objectives (Leica Microsystems S.r.l., Varese, Italy), a DFC 450C camera (Leica Microsystems S.r.l., Varese, Italy) and the Application Suite Software (Leica Microsystems S.r.l., Varese, Italy) as reported in [61]. Representative images of three independent experiments are presented in the results section.

### 2.7. Assessment of Organoids and Cell Viability via MTT Reduction

Cells and organoid viability was assessed by 3-(4,5-dimethylthiazol-2-yl)-2,5-diphenyltetrazolium bromide (MTT, Sigma-Aldrich, St. Louis, MO-IL, USA) reduction [19,20]. Briefly, MTT solution was added to the organoid cultures to a final concentration of 500 μg/mL and incubated at 37 °C, 5% CO_2_. After 2 h, medium was discarded and 20 μL of 2% SDS solution in H_2_O was added to solubilize the Matrigel. Following 2 h at 37°C, 100 μL of DMSO were added and incubated for 1 h at 37 °C to solubilize the reduced MTT. The optical density was then measured at 562 nm in a plate reader (EnSpire, PerkinElmer Italia Spa, Monza, Italy). Untreated organoids were defined as 100% viable.

### 2.8. Statistical Analyses

All data are presented as the means ± S.D. (standard deviation) of at least three experiments in triplicate (*n* ≥ 9). Statistical significance between groups was determined using Student’s *t*-test (matched pairs tests or unmatched tests were used as indicated in the figure legends). All statistical analyses were performed using JMP Software obtained by Statistical Discovery SAS Institute (*p* < 0.05, statistical significance; *p* < 0.001, high statistical significance).

## 3. Results

### 3.1. Drugs Treatment Effect on PC-3 and DU145 Cells Viability

The effect of cisplatin, paclitaxel, and docetaxel on DU145 and PC-3 cell viability was evaluated by MTT assay. Cells were exposed to eight different concentrations of each drug (the minimum and maximum doses are reported in Table 1). In Table 1, it is evident that PC-3 cells showed higher resistance to chemotherapeutic drugs than DU145 cells, in particular to paclitaxel. Consequently, the dose–response curve for each drug was performed with the highest concentrations. The toxicity test precision was described by the mean and the standard deviation of the calculated endpoint (i.e., the half-maximal inhibitory concentration IC50) from the replicates. IC50 of cisplatin, paclitaxel, and docetaxel were evaluated at 48 and 72 h in PC-3 and DU145 cells, as summarized in Table 2.

**Figure 2:**
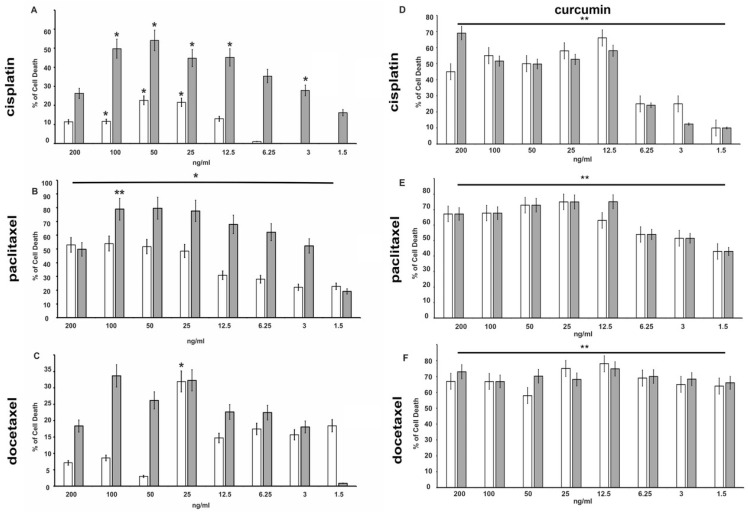
Effects of cisplatin (panel **A**), paclitaxel (panel **B**), docetaxel (panel **C**), alone or in combination with curcumin at the IC50 dose concentration (**D**–**F**), respectively) on DU-145 cells viability. Using the colorimetric MTT assay, cell death induced by chemotherapeutic drugs at the indicated concentrations was assessed after 48 h and 72 h treatment. The histograms represent the mean (% of cell death normalized to untreated control cells) of three independent experiments performed in triplicate (*n* = 9). *: *p* < 0.05, **: *p* < 0.001 compared to the controls. Gray bars indicate 48 h time, while white bars indicate 72 h time.

**Figure 3:**
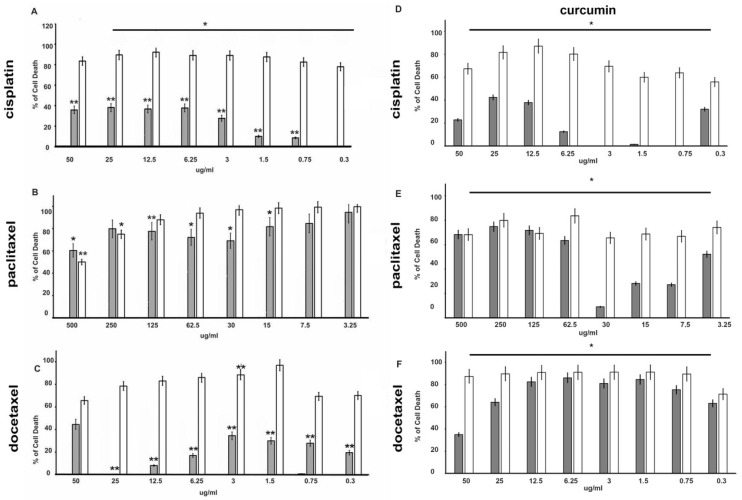
Effects of cisplatin (panel **A**), paclitaxel (panel **B**) and docetaxel (panel **C**), alone or in combination with curcumin at the IC50 dose concentration (**D**–**F**), respectively) on PC-3 cells viability. Using the colorimetric MTT assay, cell death induced by chemotherapeutic drugs at the indicated concentrations was assessed after 48 h and 72 h. The histograms represent the mean (% of cell death normalized to untreated control cells) of three independent experiments performed in triplicate (*n* = 9). *: *p* < 0.05, **: *p* < 0.001 compared to the controls. Gray bars indicate 48 h time, while white bars indicate 72 h time.

MTT experiments demonstrated that cisplatin, paclitaxel, and docetaxel exert a cytotoxic effect in a dose-dependent manner both in DU145 and PC-3 cells (Figure 2 and Figure 3).

Among the three chemotherapeutics used, paclitaxel has the greatest anti-proliferative effect on the DU-145 cell line (Figure 2B). Already after 48 h of treatment, cell death was increased by 30% in cells treated with 12.5 ng/mL (14.64 nM) reaching 50% with a dose of 25 ng/mL (29.28 nM) (Figure 2B). The effects on cell growth are more evident at 72 h of treatment. The prolonged exposure (72 h) increased the effect and 6.25 ng/mL (7.32 nM) induced over 60% of death. Even in PC-3 cells (Figure 3), treatment with paclitaxel appears more effective (Figure 3B) than other drugs tested, and already at 24 h, a percentage of dead cells of approximately 50% is reached with a dose of 7.5 µg/mL (8.78 µM). It is also interesting to note that the greatest effects occur at lower concentrations (3.25–30 µg/mL—3.81–35.13 µM), while higher paclitaxel concentration showed a lower effect, probably because the drug becomes ineffective.

A similar effect was seen in the presence of cisplatin and docetaxel in both cell lines (Figure 2 and Figure 3A,C), even if mortality does not reach the levels observed in the presence of paclitaxel.

### 3.2. Effect of Curcumin Alone or in Combination with Drugs on PC-3 and DU145 Cell Viability

**Figure 4:**
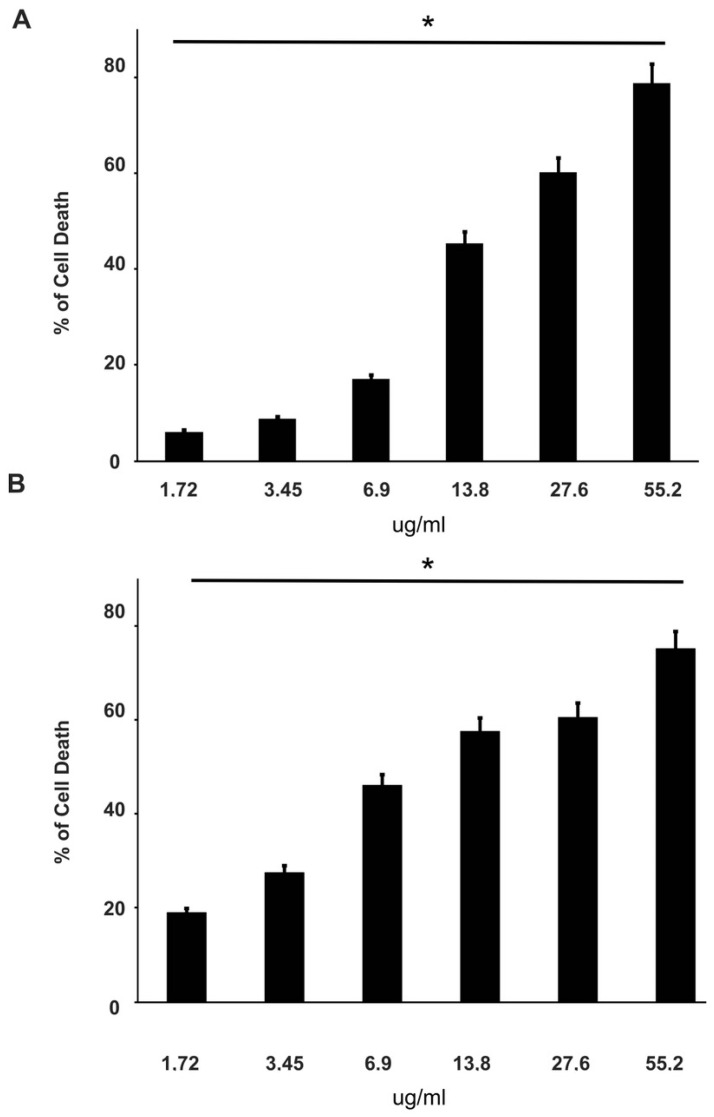
Effects of curcumin on DU-145 and PC-3 cell viability. Using the colorimetric MTT assay, cell death induced by curcumin at the indicated concentrations was assessed after 24 h in DU-145 (panel **A**) and PC-3 (panel **B**) cells. The histograms represent the mean (% of cell death normalized to untreated control cells) of three independent experiments performed in triplicate (*n* = 9). *: *p* < 0.05, compared to the control.

To evaluate the effect of curcumin on cell viability, DU-145 and PC-3 cell lines were first treated for 24 h with curcumin alone at six different concentrations between the ranges indicated in Table 1, and then in the presence of cisplatin, paclitaxel and docetaxel. Both cell lines are sensitive to curcumin, which induced cell death in a dose-dependent manner. About 80% of death is observed at 55.2 µg/mL (149.85 µM) (Figure 4).

The effects increased with prolonged stimulation over time (data not shown). To investigate the effect of curcumin in association with chemotherapeutic agents, DU-145 and PC-3 cell lines were co-treated with the indicated concentration of each drug and curcumin (IC50) for 48 h and 72 h. Interestingly, curcumin was able to enhance the effect of all chemotherapy drugs on the DU-145 cell line (Figure 2D–F). In particular, the curcumin treatment increased (from about 15% to 60%) cell death 4-fold with 12.5 ng/mL of cisplatin, and 1.5-fold (from about 35% to 50%) with 1.5 ng/mL of docetaxel even at 48 h of treatment. As expected, PC-3 cells showed high resistance to chemotherapy, in fact, curcumin in co-treatment with drugs gives an increase in cell death only in the presence of docetaxel at 48 h (Figure 3F).

### 3.3. Drugs Treatment Effects on EGFR Expression Level and ERK Activation in PC-3 and DU145 Cells

**Figure 5:**
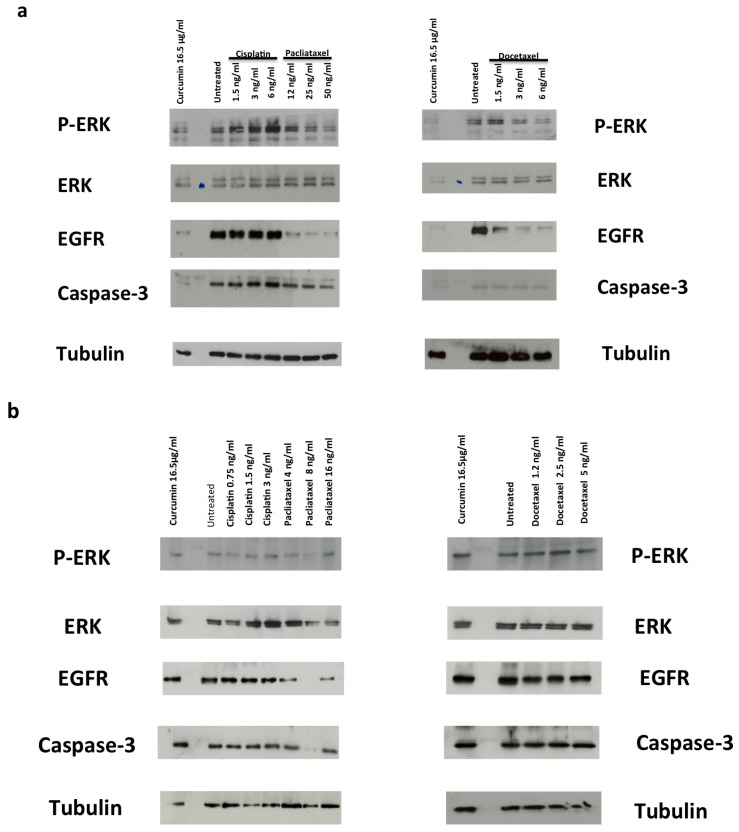
(**a**,**b**) Role of the p-ERK/ERK/EGFR in survival and apoptosis. DU-145 (**a**) and PC-3 (**b**) cells were used. In (**a**,**b**), the cells were stimulated for 48 h in the absence or presence of the indicated compounds. Curcumin was used at 16.5 µg/mL (44.79 µM). Proteins were separated by SDS-PAGE and then analyzed by Western blot, using the antibodies against the indicated proteins.

EGFR regulates cell growth, motility, differentiation, and tumorigenesis by activation of several downstream intracellular signaling pathways, including MAPK kinase and extracellular-related kinase (ERK) [62]. EGFR is frequently overexpressed in cancer, leading to uncontrolled signal transduction and to oncogenic phenotypes [63]. Accordingly, EGFR is expressed at a high level in DU145 and PC-3 cells (Figure 5). Paclitaxel and docetaxel treatment decreased EGFR expression levels while cisplatin had no effect on EGFR levels (Figure 5a,b). Moreover, paclitaxel and docetaxel reduced ERK activation (Figure 5a,b). We underline that ERK phosphorylation is related to the EGFR expression level as revealed by its evident reduction in PC cells treated with paclitaxel and docetaxel (Figure 5a,b). Curcumin induced similar results to those observed with paclitaxel and docetaxel with a greater effect in DU145 cells than in PC3 cells (Figure 5a,b).

### 3.4. CRPC 3D Organoid Size Reduction

**Figure 6:**
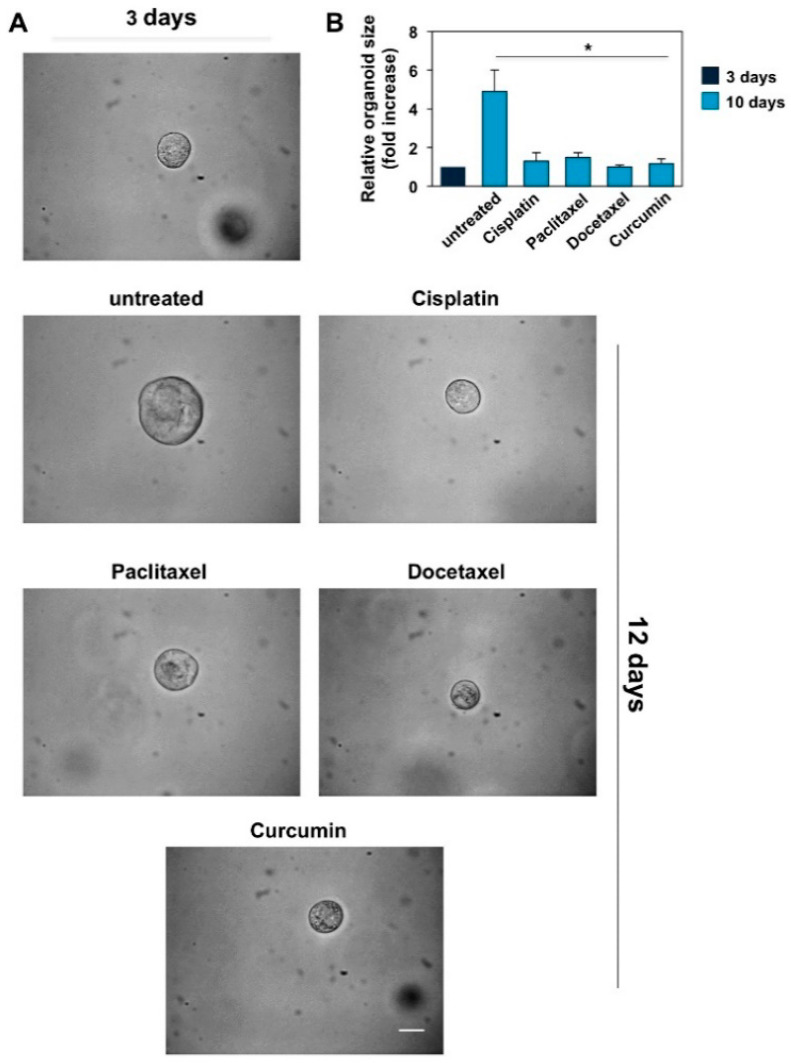
Cisplatin, paclitaxel, docetaxel (used at 25 ng/mL) and curcumin (used at 6.9 µg/mL–18.73 µM) reduce the size of DU145 cells derived organoids. (**A**): Three days after cells embedding in Matrigel, representative images were acquired as described in Method section. The 3D cultures were left untreated or treated in the absence or presence of the indicated drugs for 12 days. Were shown phase-contrast images captured on 12th day. Scale bar, 100 µ. (**B**): In the graph is represented the analysis of organoid size from DU145 cells untreated or treated with the drugs reported in the figure for 12 days. The area of organoids was calculated using Leica suite software. Three different experiments, each in duplicate, were performed and results were expressed as fold increase over the basal level analyzed after 3 days. Means and standard error of the means (SEMs) are shown, and “*n*” represents the number of the experiments. *: *p* < 0.05 for the indicated experimental points vs. the untreated controls.

**Figure 7:**
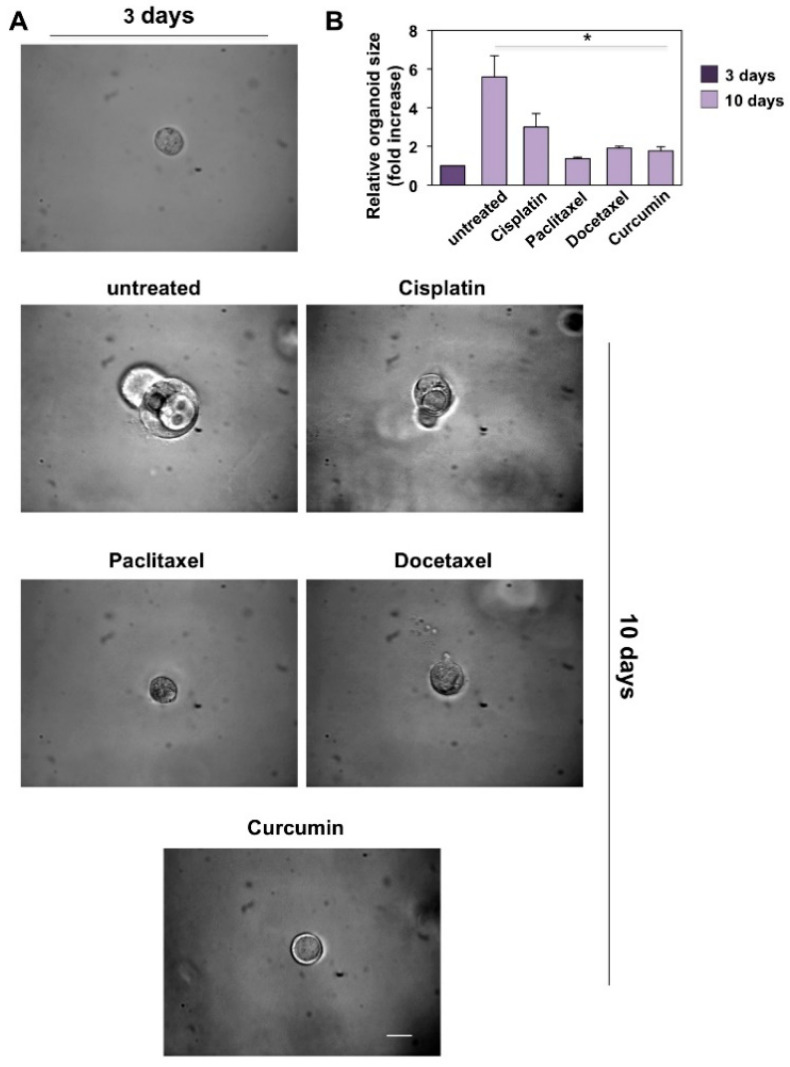
Cisplatin (used at 3.32 µg/mL–11.03 µM), paclitaxel (used at 15.6 µg/mL–18.27 µM), docetaxel (used at 20 µg/mL–24.76 µM) and curcumin (used at 6.9 µg/mL–18.73 µM) reduce the size of PC3 cells derived organoids. (**A**): Three days after cells embedding in Matrigel, representative images were acquired as described in Methods. The 3D cultures were left untreated or treated in the absence or presence of the indicated drugs for 10 days. Were shown phase-contrast images captured on 10th day. Scale bar, 100 µ. (**B**): In the graph is represented the analysis of organoid size from PC3 cells untreated or treated with the drugs reported in the figure for 10 days. The area of organoids was calculated using Leica suite software. Three different experiments, each in triplicate, were performed and results were expressed as fold increase over the basal level analyzed after 3 days. Means and standard error of the means (SEMs) are shown, and “*n*” represents the number of the experiments. *: *p* < 0.05 for the indicated experimental points vs. the untreated controls.

To recapitulate the functional and structural development of resuming PC tissue, 3D PC organoids were performed. Taking into consideration the previous data derived from MTT assays performed in DU145 and PC-3 cell lines and the necessity of monitoring the effects of the drugs in a fairly long timeframe (12 and 10 days, respectively), we chose the lowest concentration of the drugs that had an efficient inhibitory potential in 3D-model experiments. Phase-contrast images in Figure 6A and Figure 7A show that a 3D structure was observed in both CRPC cell lines used on the third day of culture in Matrigel.

The DU145 (Figure 6A) and PC-3 (Figure 7A) cell-derived organoids result in a well-differentiated and roundish phenotype [64]. On the third day of culture, organoids were untreated or treated with the drugs reported in the figures at the concentrations indicated in the relative legends. Changes in dimension and structure of organoids were monitored for 12 and 10 days for DU145 (Figure 6A) and PC3 (Figure 7A) cells, respectively. Phase-contrast microscopy images were captured and shown (Figure 6A and Figure 7A), and quantification of data was performed and graphically presented (Figure 6B and Figure 7B) as a fold increase in the relative organoid size. Data in Figure 6 show that after 12 days, organoids derived by DU145 cells increased by about 5-fold in size. Similarly, data in Figure 7 show that after 10 days, organoids derived by PC-3 cells increased by about 6-fold in size. All the drugs employed significantly reduced the organoid size after 12 and 10 days of treatment, respectively (*p* < 0.05 in Figure 6B and Figure 7B).

### 3.5. Cisplatin, Paclitaxel, Docetaxel and Curcumin Induce Organoid Disruption and Apoptosis

**Figure 8:**
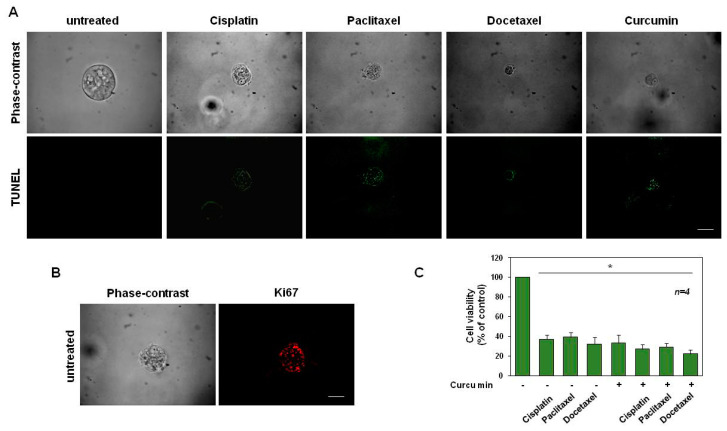
Cisplatin, paclitaxel and docetaxel (used at 25 ng/mL) and curcumin (used at 6.9 µg/mL–18.73 µM) induce apoptosis in DU145 cells. (**A**): Phase-contrast and in situ cell death KIT-stained images (green) of organoids left untreated or treated in the absence or presence of the indicated drugs for 15 days are shown. Scale bar, 100 µ. (**B**): Phase-contrast and Ki-67-stained images (red) of organoids untreated for 15 days are shown. Scale bar, 100 µ. In (**A**,**B**), images are representative of three different experiments, each in duplicate. (**C**): Cell viability was evaluated by MTT assay in DU145 organoids after 12 days of culture. The graph in (**C**), represents the organoids’ cell viability expressed as % of control, assumed as 100% of viability. Four independent experiments were carried out. Means and standard error of the means (SEMs) are shown, and “*n*” represents the number of the experiments. *: *p* < 0.05 for the indicated experimental points vs. the untreated control.

**Figure 9:**
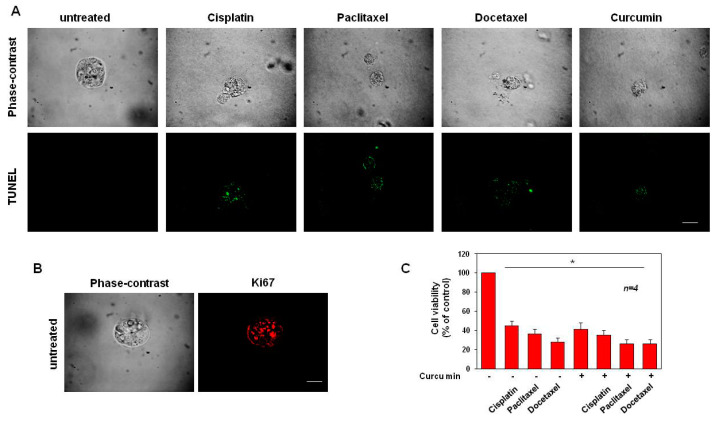
Cisplatin (used at 3.32 µg/mL–11.03 µM), paclitaxel (used at 15.6 µg/mL–18.27 µM), docetaxel (used at 20 µg/mL–24.76 µM) and curcumin (used at 6.9 µg/mL–18.73 µM) induce apoptosis in PC3 cells. (**A**): Phase-contrast and in situ cell death KIT-stained images (green) of organoids left untreated or treated in the absence or presence of the indicated drugs for 15 days are shown. Scale bar, 100 µ. (**B**): Phase-contrast and Ki-67-stained images (red) of organoids untreated for 15 days are shown. Scale bar, 100 µ. In (**A**,**B**), images are representative of three different experiments, each in duplicate. (**C**): Cell viability was evaluated by MTT assay in PC3 organoids after 10 days of culture. The graph in (**C**), represents the organoids’ cell viability expressed as % of control, assumed as 100% of viability. Four independent experiments were carried out. Means and standard error of the means (SEMs) are shown, and “*n*” represents the number of the experiments. *: *p* < 0.05 for the indicated experimental points vs. the untreated controls.

DU145 (Figure 8) and PC3 organoids (Figure 9), were exposed for 15 days to cisplatin, paclitaxel, docetaxel and curcumin at the concentrations indicated in the related legend to the figures. To investigate whether organoids with a disrupted and pointed-dark morphology in the phase-contrast microscope images (Figure 8A and Figure 9A, upper panels) corresponded to structures with increased cell death, we performed in situ cell death detection of both untreated and treated organoids. IF images (Figure 8A and Figure 9A, lower panels) showed that the drug treatments induce an increase in the number of cells positive for the green fluorescein staining, corresponding to the share of dead cells if compared to the untreated organoids, used as controls. Accordingly, Figure 8B and Figure 9B show that after 15 days of culture, untreated cells exhibit a strong positivity for Ki-67-proliferative marker (red-stained) while this immunostaining was not visible for DU145 and PC-3 organoids treated with all the drugs (data not shown).

After 12 and 10 days of treatment, DU145- (Figure 8C) and PC-3-derived organoids (Figure 9C) were used for the MTT viability assay. This approach resulted in a good, efficient, quantitative and objective way to measure organoid cell death [65]. Graphs in Figure 8C and Figure 9C show that a drastic reduction in formazan-positive organoids, compatible with a reduction of organoid cell viability, was observed after treatment with cisplatin, paclitaxel, docetaxel and curcumin, to different extents. In addition, compatibly with the results obtained in Figure 3, the curcumin was able to slightly but significantly enhance the effect of all chemotherapy drugs, on the viability reduction of DU-145-derived organoids (Figure 8C). Interestingly, unlike the data obtained in Figure 3, the cisplatin and curcumin synergistic use on PC-3-derived organoids, significantly decreased the cell viability, when compared with the cisplatin used alone. Instead, curcumin used in co-treatment with paclitaxel or docetaxel did not significantly reduce the cell viability, when compared with the drugs used alone (Figure 9C). In conclusion, these findings show that the treatment of DU145- and PC-3-derived organoids with cisplatin, paclitaxel, docetaxel and curcumin not only reduced the organoid sizes but also induced their apoptosis.

## 4. Discussion

To date, PC is one of the leading causes of cancer death in men. In patients with recurrent disease after local therapy, ADT represents one of the approaches applied to suppress tumor growth and progression [66]. However, PC patients frequently develop resistance to ADT and PC progresses towards CRPC with a high metastatic potential [67,68]. Currently, chemotherapeutic drugs improve the overall survival and disease-free survival of patients with CRPC. Multiple genes and signaling pathways are involved in the PC progression towards CRPC, but the underlying mechanisms remain poorly understood. Therefore, the discovery of new drugs to improve the therapeutic effect is relevant in CRPC treatment. In the present study, we investigated the effect of three chemotherapeutic drugs (cisplatin, paclitaxel, and docetaxel) on PC-3 and DU145 cell lines, both derived from mCRPC, comparing their effects with curcumin, as reported by Cheng AL et al. [54], in a prospective phase I clinical trial, who showed that an 8000 mg/day dose of curcumin could be safely administered to humans with minimal toxicity. In addition, it was reported the average peak serum concentrations after taking 4000 mg, 6000 mg and 8000 mg of curcumin were 0.51 ± 0.11 µM, 0.63 ± 0.06 µM and 1.77 ± 1.87 µM, respectively [54]. By now, it is well accepted that natural compounds may be a useful strategy as co-chemotherapeutic agents to improve the action of anticancer drugs in cancer management [69,70,71]. Our in vitro study reveals that the chemotherapeutic agents reduce the proliferative capacity of DU145 and PC3 cell lines in a dose-dependent manner. Between the three chemotherapeutics used, paclitaxel showed the greatest antiproliferative effect on both cell lines. Moreover, in PC-3 cells, the greatest effects occur at lower concentrations (3.25–30 µg/mL—3.81–35.13 µM), even after just 24 h of treatment. This evidence represents an essential element in reducing toxicity and side effects. Additionally, curcumin acts similarly to chemotherapeutic agents, reducing the proliferation of DU145 and PC-3 cells in a dose- and time-dependent manner.

Upregulation of EGFR and subsequent increases in ERK signaling are implicated in PC progression. Impaired endocytic downregulation of EGFR also contributes to oncogenic phenotypes such as metastasis. In this study, we investigate the effects of EGFR signaling on the ERK pathway in DU145 and PC-3 cells. Our results showed that DU145 and PC-3 cells overexpress EGFR and that chemotherapeutic agent treatment or curcumin reduced EGFR expression level and ERK signaling activation.

The main drawback in anticancer therapy development is the limited availability of biologically relevant in vitro models. As a consequence, the most relevant challenge is to develop cell culture models that resemble tumor tissues by reproducing the in vivo complex architecture more faithfully. Therefore, cancer biology, homeostasis and behavior are strongly influenced by the tumor microenvironment. Novel cell and tissue-based tests that reflect the effects of the extracellular matrix (ECM), cell–cell contacts, cell–matrix interactions, and recapitulate the tissue architecture of tumors are strongly needed. The establishment of tumor organoids and spheroids significantly increased the opportunity to investigate anti-cancer agents in vitro. New 3D cell culture techniques enable the formation of an organoid/spheroid, a miniaturized and simplified version of an organ produced in vitro that exhibits realistic microanatomy and allow cells to modulate the ECM. It is widely accepted that 3D organoids and spheroids reflect the in vivo growth of cancer cells more reliably and provide better readings for drug testing [55,64,65]. The broad spectrum of phenotypic changes observed during drug exposure can be used as a sensitive reading to measure the efficacy of the compound. Here, for the first time, we assessed the effect of chemotherapeutic agents and curcumin on spheroid models derived from DU145 and PC-3 CRPC cell lines and used a variety of methods to score their size, death and survival, by using contrast-phase, immunofluorescence microscopy approaches and colorimetric methods. Our findings show that PC-3 and DU-145 organoids were formed after three days of culture in ECM, and that cisplatin, paclitaxel, docetaxel, and curcumin, when used at different concentrations, significantly reduced the size of CRPC-derived 3D models in a relatively short time of 10 and 12 days. The detection of cell death in situ showed that the reduction observed was due to cell apoptosis. Finally, by means of the MTT assay, we observed a reduction in cell viability in organoids treated with cisplatin, paclitaxel, docetaxel and curcumin, confirming that the drugs reduce the size of organoids by inducing apoptosis and inhibiting growth. In this context, the synergistic use of curcumin and chemotherapeutic drugs decreases the cell viability of DU145- and PC-3-derived organoids.

## 5. Conclusions

PC is the second leading cause of cancer-related deaths for males and its incidence has increased significantly in recent years. The cost of PC treatment and available drugs can be high, thus limiting its potential use in less developed countries. Therefore, there is an urgent need to develop new therapeutic strategies that are safe, potent, affordable, and easy to manufacture. Several data have shown that curcumin can suppress the proliferation of both androgen-dependent and androgen-independent PC cell lines. Consequently, curcumin administration seems to be useful in PC prevention, and in co-treatment with conventional therapy to halt PC progression towards mCRPC. Despite its widely reported health benefits, the use of curcumin is hampered by its poor bioavailability which limits its clinical application. In this regard, several strategies were developed to overcome these limitations, including improving the delivery system by encapsulating curcumin in the form of nanoparticles, designing novel structural analogs, and liposomal encapsulation and emulsions. Our study provides evidence that curcumin supplementation can be used as a preventative strategy and opens up new frontiers for further studies aimed at implementing the diet with nutraceuticals. In conclusion, despite different studies being carried out to identify the potential synergistic curcumin combinations with chemopreventive/therapeutic efficacy for inhibiting PC growth, to our knowledge, this study is the first to show the ability of curcumin used alone or in combinatorial approaches to impair the size and the viability of PC-derived spheroids. However, further pre-clinical and clinical studies are needed to better understand curcumin’s mechanism of action, increased bioavailability, safety, dose efficacy and stability in order to translate curcumin as a candidate drug for PC treatment. Given the minimal toxicity and promising preclinical activity, the rationale for adding this standard care therapy would become an attractive therapeutic option for patients with mCRPC.

## Figures and Tables

**Table 1:** Drugs used to treat PC-3 and DU145 cells and the related concentrations.

	PC-3	DU145
Drug	Concentration	
DOCETAXEL	0.3–50 µg/mL 0.37–61.9 µM	1.5–200 ng/mL 1.86–247.56 nM
PACLITAXEL	3.25–500 µg/mL 3.81–585.55 µM	1.5–200 ng/mL 1.76–234.22 nM
CISPLATIN	0.3–50 µg/mL 0.996–166.06 nM	1.5–200 ng/mL 4.99–664.23 nM
CURCUMIN	1.72–55.2 µg/mL 4.67–149.85 µM	1.72–55.2 µg/mL 4.67–149.85 µM

**Table 2:** IC50 values mean for docetaxel, paclitaxel, cisplatin, and curcumin in hormone-refractory PC-3 and DU145 cancer line cells.

PC-3			DU145	
IC50 *	48 h	72 h	48 h	72 h
CISPLATIN	2.35 µg/mL 7.80 µM	1.32 µg/mL 4.38 µM	3.26 ng/mL 10.83 µM	2.17 ng/mL 7.21 µM
PACLITAXEL	13.1 µg/mL 15.34 µM	10.07 µg/mL 11.79 µM	N.D	4.42 ng/mL 5.18 nM
DOCETAXEL	9.14 µg/mL 11.3 µM	6.5 µg/mL 8.05 µM	12.5 ng/mL 15.47 nM	3.76 ng/mL 4.65 nM
CURCUMIN	8.64 µg/mL 23.45 µM	6.52 µg/mL 17.70 µM	16.5 µg/mL 44.79 µM	8.62 µg/mL 23.40 µM

* IC50: half-maximal inhibitory concentration; N.D: not determined. The IC50 of paclitaxel at 48 h could not be determined because the cells at this concentration showed drug resistance.

## Data Availability

Data is contained within the article.

## Supplementary Materials

The following supporting information can be downloaded at: https://www.mdpi.com/article/10.3390/cancers14143348/s1, Figure: The whole western blot figures.
