# Supplementary figures and images for: The Role of Curcumin in Prostate Cancer Cells and Derived Spheroids
Source: Cancers (Basel). 2022 Jul 9;14(14):3348. doi: 10.3390/cancers14143348 (PMC9320241; doi:10.3390/cancers14143348)

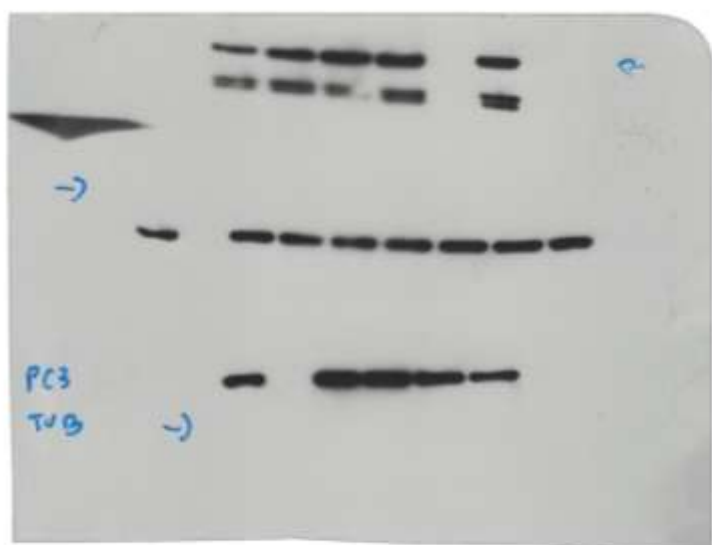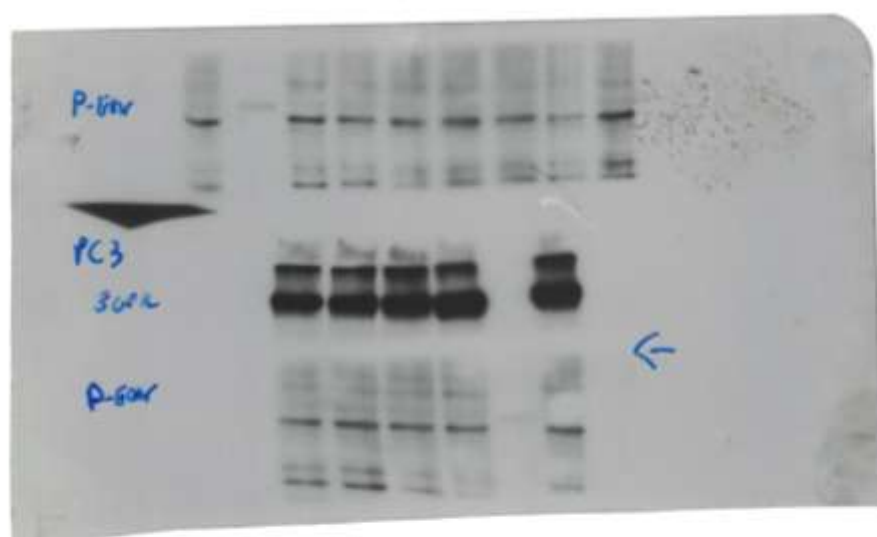

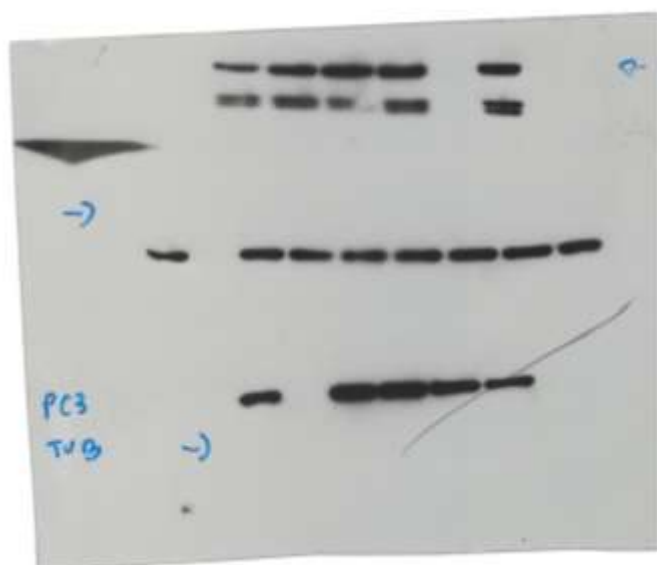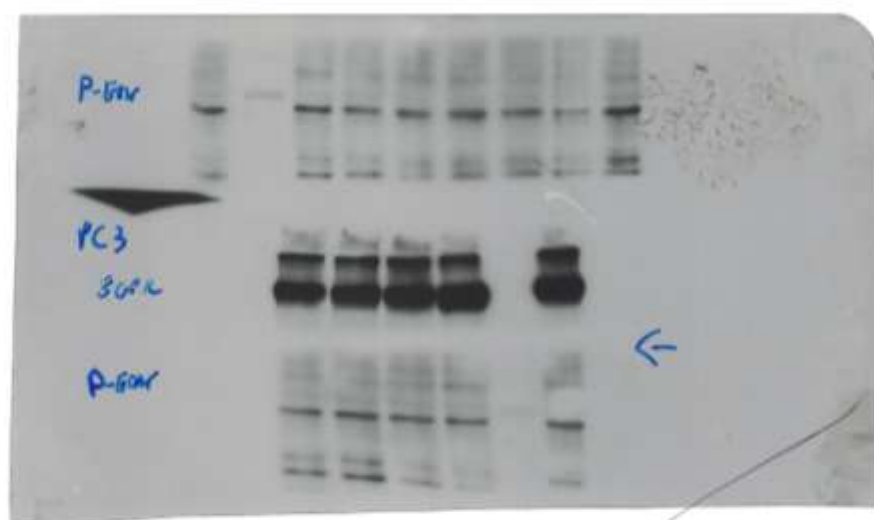

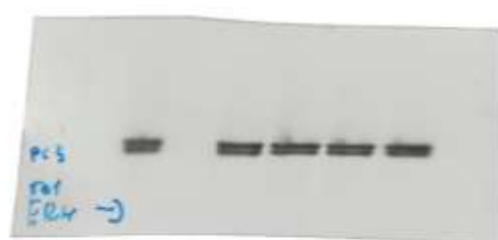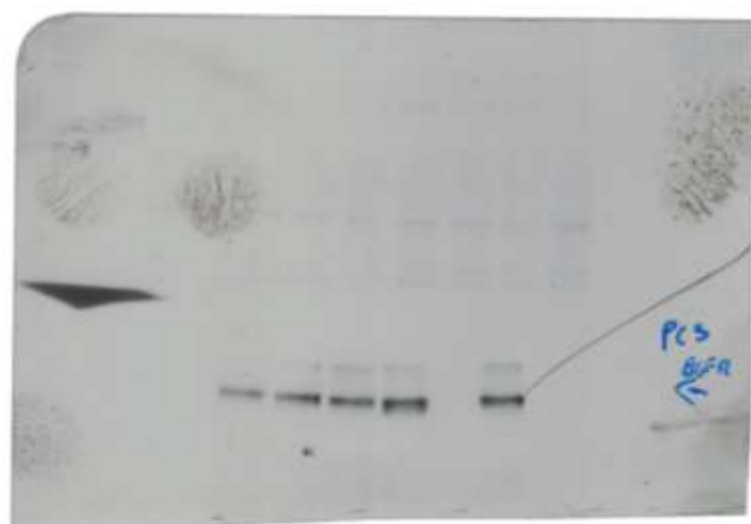

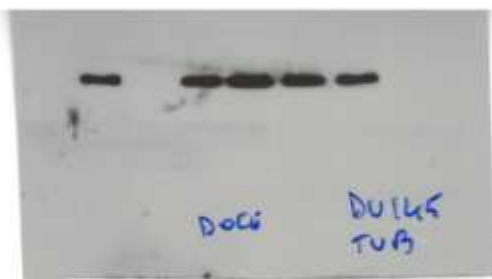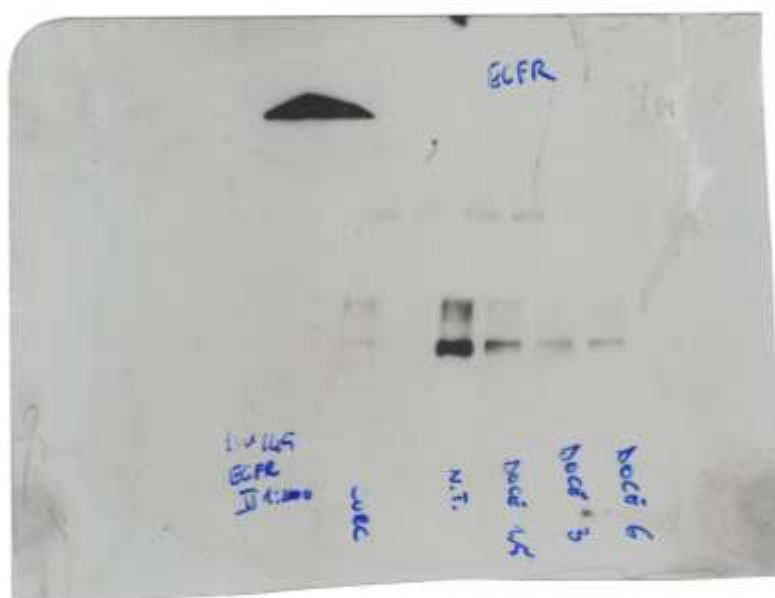



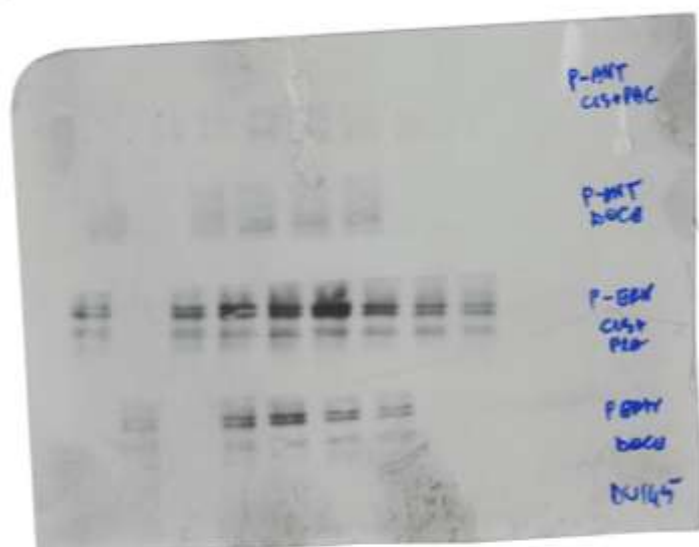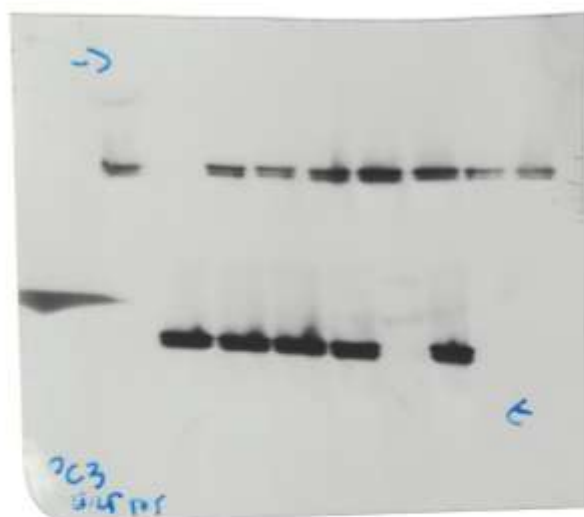

Supplement: Supplementary file 1 [file cancers-14-03348-s001.zip › cancers-1797170-supplementary.pdf]
